# Quorum Sensing Inhibitors: An Alternative Strategy to Win the Battle against Multidrug-Resistant (MDR) Bacteria

**DOI:** 10.3390/molecules29153466

**Published:** 2024-07-24

**Authors:** Helal F. Hetta, Yasmin N. Ramadan, Zainab I. Rashed, Ahmad A. Alharbi, Shomokh Alsharef, Tala T. Alkindy, Alanoud Alkhamali, Abdullah S. Albalawi, Basem Battah, Matthew G. Donadu

**Affiliations:** 1 Division of Microbiology, Immunology and Biotechnology, Department of Natural Products and Alternative Medicine, Faculty of Pharmacy, University of Tabuk, Tabuk 71491, Saudi Arabia; aam_alharbi@ut.edu.sa (A.A.A.); sh_alsharef@ut.edu.sa (S.A.); talkendy@ut.edu.sa (T.T.A.); 2 Department of Microbiology and Immunology, Faculty of Pharmacy, Assiut University, Assiut 71515, Egypt; yasmine_mohamed@pharm.aun.edu.eg (Y.N.R.); zeinab_rashed@pharm.aun.edu.eg (Z.I.R.); 3 Department of Pharmaceutical Chemistry, Faculty of Pharmacy, University of Tabuk, Tabuk 71491, Saudi Arabia; aalkhamali@ut.edu.sa (A.A.); abs_albalawi@ut.edu.sa (A.S.A.); 4 Department of Biochemistry and Microbiology, Faculty of Pharmacy, Antioch Syrian Private University, Maaret Siadnaya 22734, Syria; 5 Hospital Pharmacy, Giovanni Paolo II Hospital, ASL Gallura, 07026 Olbia, Italy; mdonadu@uniss.it; 6 Department of Medicine, Surgery and Pharmacy, Scuola di Specializzazione in Farmacia Ospedaliera, University of Sassari, 07100 Sassari, Italy

**Keywords:** quorum sensing inhibitors, MDR bacteria, antibiotic resistance, quorum quenching

## Abstract

Antibiotic resistance is a major problem and a major global health concern. In total, there are 16 million deaths yearly from infectious diseases, and at least 65% of infectious diseases are caused by microbial communities that proliferate through the formation of biofilms. Antibiotic overuse has resulted in the evolution of multidrug-resistant (MDR) microbial strains. As a result, there is now much more interest in non-antibiotic therapies for bacterial infections. Among these revolutionary, non-traditional medications is quorum sensing inhibitors (QSIs). Bacterial cell-to-cell communication is known as quorum sensing (QS), and it is mediated by tiny diffusible signaling molecules known as autoinducers (AIs). QS is dependent on the density of the bacterial population. QS is used by Gram-negative and Gram-positive bacteria to control a wide range of processes; in both scenarios, QS entails the synthesis, identification, and reaction to signaling chemicals, also known as auto-inducers. Since the usual processes regulated by QS are the expression of virulence factors and the creation of biofilms, QS is being investigated as an alternative solution to antibiotic resistance. Consequently, the use of QS-inhibiting agents, such as QSIs and quorum quenching (QQ) enzymes, to interfere with QS seems like a good strategy to prevent bacterial infections. This review sheds light on QS inhibition strategy and mechanisms and discusses how using this approach can aid in winning the battle against resistant bacteria.

## 1. Introduction

Antibiotics were discovered in the early part of the 20th century, providing humans with a potent weapon against potentially fatal microorganisms. The overuse and carelessness of antibiotic use have led to the evolution of several multidrug-resistant (MDR) strains [1,2,3,4,5].

The emergence of antibiotic resistance represents a critical issue and a big worldwide healthcare concern [6,7,8,9]. At least 1.27 million deaths worldwide and almost 5 million deaths in 2019 were directly related to antibiotic resistance. In the US, antibiotic-resistant diseases account for around 2.8 million cases annually. According to the CDC’s 2019 Antibiotic Resistance Threats Report, over 35,000 individuals have died due to antibiotic resistance [10]. The rapid evolution of antibiotic resistance in microorganisms contributes to the overconsumption of antibiotics, their widespread use in agriculture, and the lack of newly developed antibiotics [11,12,13,14,15,16,17,18,19,20,21]. Antibiotic resistance can also emerge as a result of repeated drug administration and non optimal doses used to treat dangerous infections [22,23,24,25,26,27]. If antibiotics become ineffective, infections will be difficult or impossible to treat, increasing the risk of disease spread, severe illness, disability and death [10,28,29,30,31].

This has led to a major increase in interest in non-antibiotic treatments for combating MDR bacteria; quorum sensing inhibitors (QSIs) are among these emerging alternative treatments [32,33,34,35,36,37].

Bacterial QS is a cell-to-cell communication system that generates, detects, and reacts to extracellular signaling molecules which are known as autoinducers (AIs). As the density of the bacterial population increases, AIs accumulate in the environment. The bacteria utilize this information to monitor changes in cell counts and to control gene expression as a group. Numerous processes are regulated by QS, such as motility, conjugation, antibiotic tolerance, biofilm development, sporulation, drug resistance, and the generation of virulence factors [38,39,40]. In Gram-positive bacteria, autoinducing peptides (AIPs) have been demonstrated to control QS [41,42]. In Gram-negative bacteria, A distinct class of AIs called acyl homoserine lactones (AHLs), have been demonstrated to control QS [41,43]. In addition to these two major families of AIs, it has been shown that bacteria also use a wide range of other signaling molecules, such as (i) fatty acids produced by *Burkholderia* spp., *Xanthomonas* spp., *Xylella* spp., and *Ralstonia solanacearum* [44,45]; (ii) ketones produced by *Vibrio* spp. and *Legionella* spp. [46]; (iii) AI-3, adrenaline, and norepinephrine produced by enterohemorrhagic bacteria [47]; (iv) *Pseudomonas* quinolone signal (PQS) produced by *P. aeruginosa* [48,49,50]; and (v) pheromones and short peptides produced by Gram-positive bacteria and *Bacilli* [51,52].

This review clarifies the mechanisms and strategy of QS inhibition and explores how employing this strategy can help to combat MDR bacteria.

## 2. Methodology

We searched different databases such as PubMed, Scopus, WoS, and google scholar. The search strategy utilized Medical Subject Headings (MeSH) terms and relevant keywords to capture all of the relevant literature. The following keywords were utilized “quorum sensing”, “quorum sensing inhibitors”, “antibiotic resistance”, “MDR bacteria”, “quorum sensing mechanisms”, “quorum sensing in Gram-negative”, “quorum sensing in Gram-positive”, “Possible mechanisms of QSIs”, “quorum quenching”, “application of QSIs in combating MDR”, “QSIs-NPs”, “combination of QSIs with antibiotic”, “drug repurposing”, and “clinical trial”.

## 3. Quorum Sensing: General Overview

QS is a bacterial cell-to-cell communication system that involves the creation, detection, and response to extracellular signaling molecules known as AIs. AIs accumulate in the environment as bacterial population density rises, and bacteria use these data to track changes in cell counts and collectively regulate gene expression. QS regulates several features, including sporulation, biofilm formation, antibiotic tolerance, drug resistance, conjugation, motility, and the production of virulence factors [53,54].

All known QS systems are based on three fundamental concepts, regardless of variations in their regulatory elements and molecular mechanisms. Firstly, the members of the bacterial community produce AIs. At a low cell density (LCD), AIs diffuse away, thus their concentrations are lower than the detection threshold. On the other hand, at a high cell density (HCD), the cumulative creation of AIs results in high local concentration, allowing for detection of and response to these signals [53,54]. Secondly, AIs are detected by cytoplasmic or membrane-based receptors. Thirdly, the identification of AIs triggers the synthesis of additional AIs besides stimulating the expression of genes required for collaborative actions. This feed-forward autoinduction loop supposedly improves population synchronization [55,56].

Collectively, when the concentration of AIs exceeds a given threshold with the bacterial population density, the production of certain specific genes can be initiated to regulate the bacterial population adaptability [57].

## 4. Initial Explanations of the Quorum Sensing Mechanism

The current state of information regarding the control of *S. pneumoniae*’s genetic competence was first published in the mid-1960s and early 1970s. This research shed light on the process of bacterial cell communication and the modulation of luminescent bacterial activity [58,59]. Then in the 1980s, significant studies on *V. fischeri*’s luminescent genes and the autoinducer N-3-oxohexanoyl-L-homoserine lactone 3OC6-HSL were published [60,61]. The term “quorum sensing”, was first used in a review paper by Fuqua et al. [62].

### 4.1. Quorum Sensing System in Gram-Negative Bacteria

Different bacterial pathogens can express QS systems; among Gram-negative bacteria, *P. aeruginosa* [63], *E. coli* [64], and *A. baumannii* [65] have been reported to express QS systems. Table 1 describes Gram-negative bacteria’s QS system and associated phenotypes [66].

**Figure 1:**
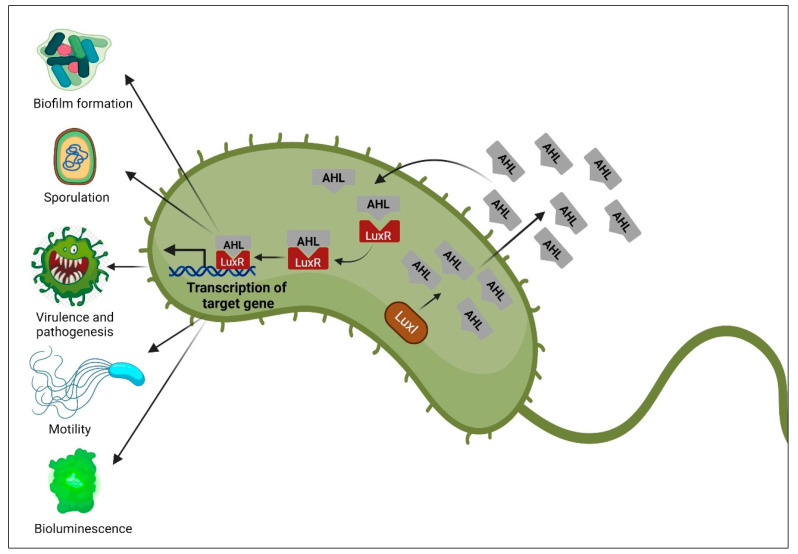
QS mechanism in Gram-negative bacteria: based on the production of N-Acyl homoserine lactone (AHL) autoinducer from LuxI, which is then attached to the LuxR receptor to trigger the transcription of the target gene [69]. Created with BioRender.

N-Acyl homoserine lactone (AHL) is responsible for signaling in Gram-negative bacteria and was initially discovered in the marine bacterium *V. fischeri*. LuxI/LuxR-type QS occurs in Gram-negative bacteria via different operons. LuxI proteins are responsible for the production of signaling molecules, particularly for the AHL, whereas LuxR attaches to AIs and initiates the transcription of the target gene as soon as they reach a specific threshold (Figure 1) [65]. These proteins create a unique AHL for each bacterial spp. [66]. AHL changes according to the length of the carbon chain [67]. Notably, AHL is not synthesized by *E. coli* and *Salmonella* spp. because they lack the LuxI protein. However, they both produce the SdiA protein, which identifies and attaches to AHL made by other bacteria [68].

Four QS systems were identified for *P. aeruginosa*: Las, Rhl, pseudomonas quinolones signal (PQS), and integrated QS (IQS). The first two are AHL-dependent QS systems [70,71].

A lactone synthase enzyme (LuxI) catalyzes the synthesis of AHL autoinducers in the las and rhl systems. These systems include the synthesis of C4-HSL, 3OC12-HSL, and two molecules of AHL [72] and consequently, the expression of multiple genes linked to the synthesis of biofilm, proteases, exotoxins, rhamnolipids, and pyocyanin [73].

### 4.2. Quorum Sensing System in Gram-Positive Bacteria

**Figure 2:**
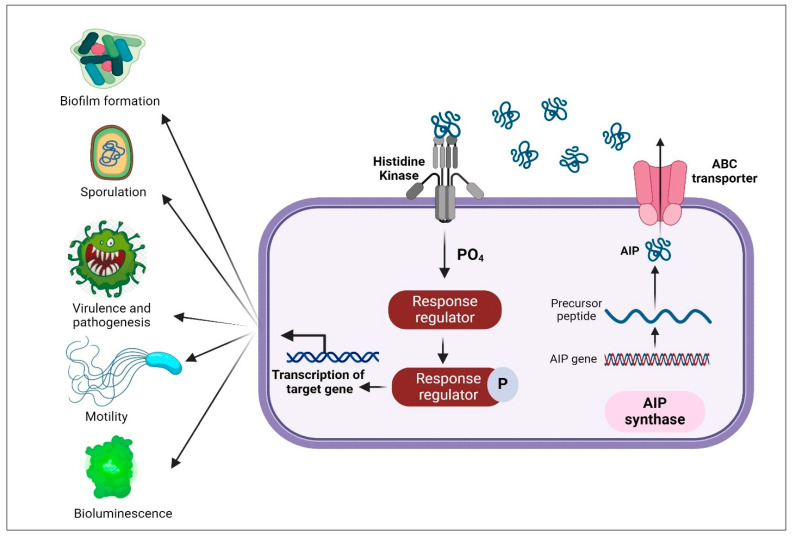
QS in Gram-positive bacteria: it involves the synthesis of autoinducer peptide (AIP) which is carried into the bacterial cell by the ABC transporter and binds to histidine sensor kinase segment; once activated, it auto-phosphorylates, and triggers targeted gene expression [69]. Created with BioRender.

QS regulation in Gram-positive bacteria is typically mediated by the autoinduction of cyclic peptides, with phosphorylation cascades acting as the signaling mechanism [38]. Since peptides cannot pass through the membrane of a bacterial cell, they typically need a transporter to export them to the extracellular environment (Figure 2) [74]. Numerous bacteria, including *S. aureus* [38], *S. pneumoniae* [75], *C. botulinum* [76], and *B. subtilis* [77] have this QS mechanism and have the ability to control various phenotypes (Table 2) [66].

**Table 1:** Gram-negative bacteria’s quorum sensing system and associated phenotypes [66,78,79,80].

Microorganism	QS	Regulated Phenotypes	Ref.
*P. aeruginosa*	*Las*, *Rhl*, *PQS*, *IQS*	Factors contributing to virulence include elastase, alkaline protease, rhamnolipids, pyocyanin, pyoverdine, motility, and biofilm formation.	[70]
*V. fischeri*	*LuxI*, *Ain*, *LuxS*	Motility, colonization within the host, and bioluminescence expression	[38]
*E. coli*	*SdiA*	Biofilm formation and motility	[64]
*A. baumannii*	*Lux*, *abaI/abaR*	Biofilm formation and motility, growth characteristics, and morphology	[65]
*Legionella*	*LqsA-LqsR*	The formation of a transmissive *L. pneumophila* Subpopulation at the *Legionella*-containing vacuole (LCV) periphery and phenotypic heterogeneity.	[78]
*B. cepacia*	*CepI/CepR*	Siderophore and protease production	[81]
*P. aureofaciens*	*PhrI/PhrR*	phz (phenazine antibiotic biosynthesis)	[82]
*M. xanthus*	*SasSRN*	sporulation	[83]

**Table 2:** Gram-positive bacteria’s quorum sensing system and associated phenotypes [66,79,80].

Microorganism	QS	Regulated Phenotypes	References
*S. aureus*	*Agr*	Synthesis of nucleases, lipases, and proteases	[84]
*S. pneumoniae*	*LUX*	Autolysis and biofilm formation	[75]
*C. botulinum*	*Agr*	Sporulation and Production of botulinum toxin	[76]
*B. subtilis*	*ComQXPA*	Production of biofilm and surfactin	[77]
*C. maltaromaticum*	*AMP*-like peptide pheromone *(CS)*	Synthesis of Class II bacteriocin	[85]
*C. piscicola*	*AMP*-like peptide pheromones (*CbnS*, *CbaX)*	Synthesis of Class II bacteriocin	[86,87]
*E. faecalis*	*GBAP*, *FsrB*, *CyIL AMP*-like peptide pheromone *(EntF)*	Synthesis of Class II bacteriocin	[87,88]
*L. plantarum*	*LamD558 AMP*-like peptide pheromone *(PlnA)*	Synthesis of exo-polysaccharides synthesis, cell membrane proteins	[87,89,90]
*L. sakei*	*AMP*-like peptide pheromone *(SppIP)*	Synthesis of Class II bacteriocin	[87,91]
*L. lactis*	Nisin	Synthesis of lantibiotic	[87]
*S. mutans*	*CSP (ComC) XIP (ComS)*	Synthesis of bacteriocins and biofilm	[92,93]

## 5. QS Inhibition

Bacteria that can identify this QS communication have the ability to obstruct it at various phases. Numerous QSIs have been documented to function through various mechanisms: (i) inhibiting the synthesis of signal molecules; (ii) breaking down signal molecules through enzymatic degradation; (iii) competing with signal molecules for binding to receptor sites; (iv) blocking the binding of signal molecules to gene promoters and inhibiting the expression of genes; and (v) scavenging AIs by antibodies and macromolecules like cyclodextrins [1,94,95,96,97,98]. Prokaryotes produce a variety of enzymes that can break down QS signal molecules, or AHLs in particular. These enzymes include acylases, lactonases, and oxidoreductases [99]. Numerous small molecules have also been reported to function as QSIs, including dicyclic peptides, noncognate AHLs, and some intermediates of the AHL biosynthesis pathway [100,101]. It is known that many different types of plants can produce QSIs, which can either compete with signal receptors or degrade QS signals [102,103,104,105]. It has been reported that extracts from various edible plants and fruits, such as grapefruit, *Medicago truncatula*, *Curcuma longa*, cinnamon, and *Emblica officinalis*, are effective against infections brought on by plant pathogens [1,102,103,106,107,108,109,110].

## 6. Standards for Choosing QSIs

According to reports, an effective QSI needs to meet at least some of the following criteria: (a) small size, capable of effectively reducing the expression of QS-regulated genes; (b) high specificity for a particular QS regulator; (c) chemical stability and resistance to degradation by different host metabolic systems; and (d) ideally longer than the native AHL. Due to QSI’s characteristics, the bacteria are unlikely to develop resistance to the drug(s) used to treat the infection. Additionally, these compounds are unlikely to have an impact on the population of beneficial bacteria that colonize the host [111]. Eventually, it is anticipated that these QSIs will speed up the search for treatments for infectious disorders because they lack antigenicity due to their small molecular weights [1].

## 7. Possible Mechanisms of QSIs

### 7.1. Targeting AIs Biosynthesis

A key factor in QS inhibition and limiting the development of pathogenic symptoms is reducing cell-to-cell communication and hindering the formation of these connections.

In terms of QSI design and development, focusing on the synthesis of universal signal molecules is a reasonable approach. The precursor S-adenosyl-homocysteine (SAH) is converted into the AI-2A signal molecules by a sequence of two distinct enzymes [112]. *LuxS*/AI-2 plays a major role in the growth characteristics, biofilm formation, virulence factor production, and metabolism of different bacterial strains [113]. Prokaryotes are known to have the *luxS*/AI-2 QS system, which is present in roughly half of all sequenced bacterial genomes. Although bacteria react differently to various forms of AI-2, *LuxS* synthase is responsible for synthesizing the majority of AI-2 forms [114]. Moreover, both Gram-positive and Gram-negative bacteria use the *LuxS* enzyme for signaling [115]. Thus, *LuxS* presents itself as a promising candidate for the development of inhibitors capable of preventing QS in a variety of bacteria [116].

Furthermore, *mammalian* spp. lacks the *LuxS* enzyme, which lowers the possibility of inhibitors having unintended off-target effects on host cells [112]. Several peptides that can bind to the *LuxS* enzyme in Streptococcus were used to test the inhibition of *LuxS* [117]. Another study demonstrated that certain peptides could bind *LuxS* and partially block its function. For the best bacterial pathogenicity in *Edwardsiellosis*, a fish disease brought on by the Gram-negative pathogen *Edwardsiella tarda*, *LuxS*/AI-2-based QS systems were necessary [118]. The plasmid p5906 encodes peptide 5906, which was employed as a broad-spectrum antibacterial QSI that inhibited *LuxS* activity by unidentified mechanisms [119].

The biocompatibility and intestinal adaptation of probiotic lactic acid bacteria such as *L. sakei* NR28, made them attractive candidates for assessing their AI-2 inhibitory capacity against pathogenic bacteria [120]. The LuxS/AI-2 signaling system is essential for controlling numerous virulence mechanisms of the foodborne pathogen enterohaemorrhagic *E. coli* O157:H7 (EHEC), which causes hemorrhagic colitis [120,121]. The antimicrobial activity of *L. sakei* NR28 against *E. coli* O157:H7 was assessed, and the findings support the idea that *L. sakei* slightly reduced *E. coli* viability.

Nevertheless, transcriptional analysis employing a co-culture of *L. sakei* and *E. coli* verified that *L. sakei* NR28 decreased the luxS AI-2 synthase QS regulator genes [120]. Furanone compounds such as furanone (5Z)-4-bromo-5-(bromomethylene)-3-butyl-2(5H)-furanone, which is present in the marine algae *Delisea pulchra*, can inhibit AI-2 QS in *E. coli* [122].

The AI-2 signaling molecule is phosphorylated by the kinase enzyme *LsrK*. Consequently, *LsrK* inhibition would result in QS inactivation and stoppage of virulent factor production [123]. The QS cascade is activated when the phosphorylated form of AI-2 binds to transcriptional repressor protein (LsrR), causing it to be released from the promoter of Lsr operon [123]. Certain genes cannot start to express when AI-2 is not phosphorylated by *LsrK* because it hardly binds to the repressor. So, *LsrK* is considered a potential target for QS inhibition, because phosphorylation is the vital step for displacing the repressor and the activation of QS pathways [116]. In a recent study, a fast assay was developed to check for the inhibitory activity of 91 compounds against *LsrK* and QS disruption. According to the results, all of these compounds were active against *LsrK* kinase and deactivating QS [124]. To assess their efficacy as *LsrK* inhibitors, researchers in a different study created a library of AI-2 precursor molecules or (S)-4,5-Dihydroxy-2,3-pentanedione (DPD) analogs and found that the pyrazole moiety was the crucial structural component that inhibited *LsrK* [125]. (Table 3) provides an overview of additional representative studies on the targeting of signaling molecules and the AI-2 universal signal.

**Table 3:** An overview of different mechanisms of QSIs through targeting the biosynthesis of AI [116].

Bacteria	QSI	Mechanism	Ref.
*P. aeruginosa*	Molecularly imprinted polymers (MIPs)	Interfering with N-(3-oxododecanoyl)-L-homoserine lactone (3-oxo-C12AHL) autoinducers	[126]
	AHL-lactonase AiiA	Degradation of AHL signaling molecules	[127]
	Boronic acid derivatives	Reduction in signal molecule production	[128]
	3-Hydroxy-2-methyl-4(1H)-quinolone 2,4-dioxygenase	Decrease in the PQS-signal molecule expression	[129]
*S.suis* serotype 2	TNRHNPHHLHHV (peptide)	Binding to *LuxS* enzyme and inactivate production of AI-2 signals	[117]
*S. pneumoniae*	Sinefungin	LuxS downregulation to inhibit the synthesis of AI-2	[130]
	Using *S. pneumoniae LuxS* mutant strain	Use of LuxS/AI-2 mutant strain that lacks *LuxS*/AI-2 signalling	[75]
*E. coli (AK-117)*	CRISPRi	Suppression of *LuxS* synthase and AI-2 synthesis	[131]
*B. cepacia*	Diketopiperazines	disrupting the signal	[132]
*E. carotovora* and *P. fluorescens*	Hexanal	Lowering AI-2 Signals	[133]
*H. parasuis*	The *LuxS* mutant strain of *H. parasuis*	Utilizing the *LuxS*/AI-2 signaling mutant strain of *H. parasuis*	[113]

### 7.2. Preventing the Production of AHLs in Gram-Negative Bacteria

Inhibiting the synthesis of signal molecules like AHL molecules is another quorum quenching (QQ)-relevant tactic. The typical components of an AHL signal molecule are a fatty acyl side chain, which can range in length from 4 to 18 carbons depending on how saturated it is, and a homoserine lactone ring. SAM, acyl-ACP substrates, and acyl-HSL synthases, which are enzymes in the *LuxI* family, are the sources of AHL signals. Therefore, it was proposed that inhibiting the synthase enzyme or focusing on SAM and acyl-ACP biosynthesis could reduce the production of AHL [112]. This method has an advantage because the enzymes needed to produce inducers are not found in mammalian cells but are encoded in the genomes of many bacteria [134].

HdtS, LuxI, and LuxM are the three enzymatic systems that generate the AHL synthase enzyme; nevertheless, Lux I-type synthase is the most common target of these enzymes [135]. Structural analogs of S-adenosyl-methionine (SAM) are the most researched AHL synthase inhibitors. Sinefungin, L/D-S-adenosyl homocysteine, and butyryl-SAM have been shown to inhibit QS singling’s first step and reduce AHL synthesis in pathogenic *P. aeruginosa* in vitro [136]. SAM is a crucial constituent of various enzymes in biological systems, and its analogs have the potential to impede the production of AHL [137,138]. For example, all components of the bacterial methyl cycle were decreased when *E. coli* was grown in a methionine-free environment [139]. Since the AHL signal is made of carbon, which comes from the host’s fatty acid biosynthesis intermediates, its production is impacted by the absence of methyl cycle components [40].

### 7.3. Preventing the Synthesis of AIPs in Gram-Positive Bacteria

Studies on blocking AIPs are scarce because AIPs are synthesized by ribosomes and peptidase enzymes are also necessary for the growth and survival of bacteria. Their inhibition is therefore bactericidal and aids in developing bacterial resistance [112]. However, a great deal of research has been carried out to understand how signals work, ultimately resulting in the creation of tactics for inhibiting these signals [138,140].

### 7.4. Targeting the AI-2 Synthases

By targeting the LuxS enzyme, which breaks down S-ribosyl-L-homocysteine (SHR) into 4,5-dihydroxy-2,3-pentanedione (DPD) and L-homocysteine, it may be possible to interfere with AI-2 and lessen the development of microbial pathogens. The ability of L-homocysteine and S-homoribosyl-L-cysteine to inhibit *LuxS* synthases and SHR hydrolysis in a variety of bacterial spp. led to their approval [57]. Moreover, it has been shown that a variety of naturally occurring brominated furanones inhibit the LuxS enzyme in a concentration-dependent manner [141]. To determine which brominated furanone combinations can inhibit QS processes in the *V. harveyi* reporter strain, a variety of combinations have been synthesized and assessed [142]. Since *V. harveyi* lacks the AHL signal receptor, it can only react to cross-bacterial communication via the AI-2 signal. It has been demonstrated that the presence of furanone derivatives decreases the bioluminescence in *V. harveyi*, suggesting a potential function for these compounds in interfering with bacterial communication. Furanone also decreased *S. epidermidis*’ ability to form films by 57% [142]. Isolated surfactin from *B. subtilis* is one of the natural compounds that may target the LuxS/AI-2 QS system and inhibit biofilm formation by 70% at 1/2 MIC (16 µg/mL) [143].

### 7.5. Targeting AI Receptors

Reducing bacterial virulence and infection can be achieved by either competing for receptors or inactivating receptors in QS signaling (Table 4) [116]. It has been discovered that two classes of QS inhibitors, flavonoids and furanones, can bind to the receptors of different pathogenic bacteria [144,145]. The first known QS inhibitors were halogenated furanones, which are generated by the marine macroalga *Delisea pulchra*. Their mechanism of action is based on competitive binding to LuxR receptors [146]. Research has shown that the binding of flavonoids to QS receptors can significantly reduce the production of *P. aeruginosa* virulence factor [144].

Additionally, sitagliptin, a medication used to treat type 2 diabetes, can interact with the receptor LasR in *P. aeruginosa*. Biofilm formation is significantly inhibited by even a small inhibitory concentration of sitagliptin [147]. Furthermore, a few QS inhibitors can bind to several receptors at once. An example of a QS inhibiting agent produced by *Lactobacillus* is 3-benzene lactic acid (PLA), which binds to receptors RhlR and PQS R more strongly than it does to its cognate ligands BHL and PQS in *P. aeruginosa* [148]. Natural substances like piperine and embeline can prevent *S. mutans* from producing biofilm by deactivating the receptors involved in QS pathways [149].

Structural analog to AHL can reduce the production of elastase, rhamnolipid, and other virulence factors by blocking the receptors in *P. aeruginosa*. N-decanoyl-L-homoserine benzyl ester, thiolactones, and other compounds are examples of analogs [150,151,152]. In certain situations, QS receptor antagonists reduce the therapeutic dosage of antibiotics while increasing their activity [153]. When administered at subinhibitory doses, several aminoglycoside antibiotics exhibit anti-virulence and antibiofilm properties through their binding to QS regulatory receptors [154]. By directly attaching to LasR receptors, the plant flavonoid naringenin inhibits the synthesis of virulence factors that are regulated by QS [155].

Some QS inhibitors, like meta-bromo-thiolactone, can also directly deactivate the receptors to stop the production of virulence factors and the formation of biofilms [156].

Many QSIs block the function of bacterial signal receptors, preventing cell-to-cell communication and reducing the pathogenicity and virulence of infectious bacteria by creating an inactive receptor–signal complex. The most obvious tactic is to target AI signal receptors; however, not all of the listed QSIs have been identified as agents that disrupt AI receptors [137].

**Table 4:** An overview of different mechanisms of QSIs through targeting AI receptors. Modified from [116].

Bacteria	QSI	Mechanism	Ref.
*P. aeruginosa*	Morin (3,5,7,2’,4’-Pentahydroxyflavone	Inhibition of the receptor RhlR and LasR	[157]
	Meta-bromo-thiolactone	Inhibition of the receptor RhlR and LasR	[140]
	Flavonoids	LasR and RhlR receptors’ antagonistic interactions	[144]
	V-06-018	LasR receptor antagonistic interactions	[158]
*P. aeruginosa and E. coli*	Thiolactone derivatives	LasR receptor’s antagonistic interactions	[159]
	PQS R antagonist	PQS R receptor’s antagonistic interactions	[159]
*P. aeruginosa and B. cenocepacia*	3-Azidodihydrofuran-2(3H)-one (2)	AHL analogs	[160]
*P. fluorescens*	Cinnamaldehyde	LasR receptor’s antagonistic interactions	[161]
*K802NR* strain of *E. coli*	Steviol glycosides and aglycon steviol	LasR receptor’s antagonistic interactions	[162]
*A. hydrophila*	Vanillin (4-hydroxy-3-methoxy benzaldehyde)	AHL receptor interference	[163]
*C. violaceum CV026* *C. violaceum* and others	2(5H)-Furanone	Interfering with different AHL signaling molecules	[164]
	Senegalia nigrescens	directing QS signal binding to the appropriate receptors	[161]
*F. nucleatum*	Brominated furanone derivative	Antagonist of the AI-2 signal	[165]
*S. mutans*	Extracts of embelin and piperine	suppressing the QS-related receptors’ activity	[149]
*L.monocytogenes*	Furanone derivative	removing QS signaling molecules from their corresponding receptors	[166]

### 7.6. Targeting the AHL Receptors on Gram-Negative Bacteria

The most prevalent AI receptor protein in Gram-negative bacteria is called LuxR-AHL. As a result, targeting this complex with specific QSIs is a useful backup plan for managing pathogenesis. The synthesis and design of inhibitors such as AHL analogs, structurally independent AHLs, and naturally occurring QSIs are the foundation for strategies aimed at breaking the link between the signal receptor protein and AHLs [167].

#### 7.6.1. AHL Analogs

The chemical structure of AHL analogs (lactone ring and acyl side chain) is the main factor influencing cell-to-cell communication via AHL signals. So, any change in their structures such as the incorporation of any functional group in the acyl side chain or changes in chirality and geometry blocks the interaction between the receptor and signal. For example, when the active methylene group was inserted into the AHL, the protein-signal binding of the receptor was lowered by 50%, and the activity was reduced by 90% when a second methylene group was introduced. Hence, using AHL analogs is a highly successful tactic to regulate processes with QS signals [1]. In *P. aeruginosa*, *A. tumefaciens*, and *V. fscheri*, respectively, several bulky groups that may inhibit the LasR, TraR, and LuxR receptors have been added to the acyl side chain to produce specific AHL analogs [168].

#### 7.6.2. Structurally Unrelated AHLs

The actual application of structurally unrelated AHLs in vivo is still very limited, even though a 90% loss of binding capacity was achieved in vitro. More structurally unrelated AHL signals as alternative compounds need to be found. It has been shown that some antibiotics such as azithromycin, doxycycline, and ciprofloxacin; some β-lactams, such as ceftazidime, cefepime, and imipenem, at a sub-inhibitory concentration; cefoperazone and its metallic derivatives; and various synthetic furanones can inhibit QS signaling and decrease virulence factors like motility and film formation in some Gram-negative bacteria [169]. Furthermore, the effectiveness of nonsteroidal anti-inflammatory drugs (NSAIDs) like meloxicam and piroxicam was approved as QSIs [170]. Interestingly, nanoparticles (NPs) have been reported to reduce the pathogenicity of certain bacteria. For example, the Qs signaling system of *P. aeruginosa* was inhibited by copper ion NPs and silver NPs [171,172], and *V. cholerae* was inhibited by gold NPs [173].

#### 7.6.3. Natural Analogues of QS Inhibitors

Numerous natural compounds possess the ability to impede the QS signaling pathways of specific microorganisms.

For instance, limonene from *Citrus reticulata* inhibited the production of AHL signaling in *P. aeruginosa* by 33% and biofilm formation by 41% at 0.1 mg/mL [174]. Furthermore, the identical substance was extracted from *Eucalyptus radiate* and demonstrated the ability to interfere with QS-regulated pyomelanin pigment synthesis in *A. baumannii* [175]. Similarly, *S. marcescens*’ swarming motility and biofilm formation were inhibited by the phenolic extract of *Rubus rosifolius* [176]. Flavonoid-rich fraction of *Centrella Asiatic* inhibited *P. aeruginosa* PAO1’s synthesis of pyocyanins, formation of biofilms, swarming motility, elastolytic, and proteolytic activities in a concentration-dependent manner [177]. The methoxyisoflavan compound that was isolated from *Trigonella stellate* inhibited the formation of pyocyanin, protease, hemolysin activity, and biofilm formation in *P. aeruginosa* and decreased the production of violacein in *C. violaceum* [178]. Biofilm formation of *E. coli* and *P. aeruginosa* was inhibited by *Buchanania lanzana* Spreng’s methanolic extract [179]. *Amomum tsaoko* (Zingiberaceae) ethanol extract decreased the biofilms of foodborne pathogens *S. typhimurium*, *S. aureus*, and *P. aeruginosa* by 51.96%, 47.06%, and 45.28%, respectively [180]. Garlic extract may lessen *P. aeruginosa* infections by inhibiting their signaling system and reducing the formation of their biofilms [181]. Likewise, water-soluble cranberry extracts prevented *V. cholerae* from forming biofilms [182]. Additionally, it made people more susceptible to some antibiotics such as azithromycin and tobramycin as well as to neutrophil-mediated phagocytosis [183].

### 7.7. Histidine Kinase Receptor Targeting in Gram-Positive Bacteria

Gram-positive bacteria have two QS systems under regulation: a membrane-attached histidine kinase receptor and a reactive transcriptional regulator [184]. Currently, the action of the Gram-positive bacterial receptor and pathogenesis can be inhibited by specifically targeting these receptors with AIP antagonists [112]. For instance, four different thiolactone peptides (AIP I–IV) are used by the *S. aureus* agr system, an AIP-mediated QS system, to regulate bacterial behavior [137,185].

### 7.8. Targeting LuxP Receptors

There are currently three identified protein receptors that mediate AI-2 signaling. The *LuxP* gene in *V. harveyi* is the first, the *LsrB* gene in *S. typhimurium* is the second, and the *RbsB* gene in *Aggregatibacter actinomycetemcomitans* is the third [186]. The development of substances that can bind to these receptors offers a promising method of inhibiting the AI-2 QS system’s functions [112,187]. The sulphone compound, for example, had an antagonistic effect on *V. harveyi* LuxP receptors [188]. At sub-MIC concentrations, specific aromatic groups like polyols and phenylboronics were also found to inhibit the production of bioluminescence by *V. harveyi* [103].

### 7.9. Enzymatic Inactivation of AIs

A promising tactic is the enzymatic inactivation or degradation of extracellular antimicrobial peptides, which can significantly lower microbial resistance without putting the bacterial cell under stress [167]. The QQ enzymes are divided into two classes: class I comprises the enzymes that hydrolyze AHL molecule by deactivating the lactone rings, such as AHL-lactonase, AHL-acylase, and paraoxonase (PON), while class II comprises oxidoreductases, which convert carbonyl to hydroxyl [189].

#### 7.9.1. Lactonase Enzyme

**Figure 3:**
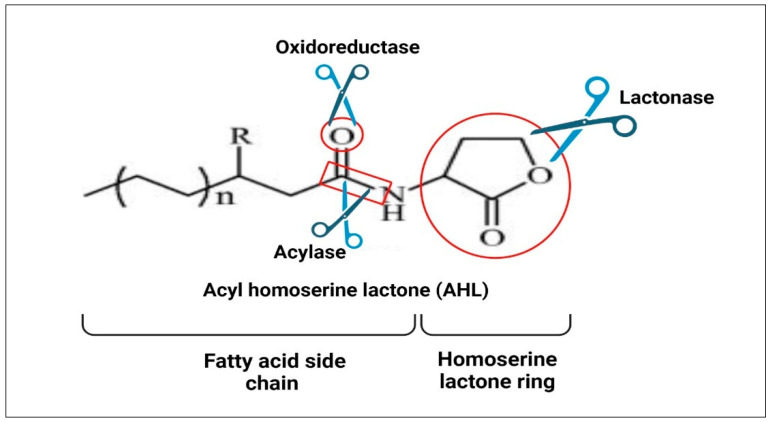
Auto-inducers’ enzymatic inactivation by the enzyme’s lactonase, acylase, and oxidoreductase. Created with BioRender.

The lactonase enzyme works by hydrolyzing the ester bond of the homoserine lactone ring (Figure 3). *Bacillus* spp. that was isolated from soil was the first microbe documented to produce the lactonase enzyme [190]. This enzyme exhibits the broadest spectrum of AHL specificity and can break down all signals, irrespective of the length or substitutions of the acyl-side chain [191]. In mammalian tissues, the lactonase enzyme has also been identified as PONs, which fall into three categories: PON1, PON2, and PON3. The genes *PON1* and *PON3* have been identified in the liver and kidneys, and the blood circulation linked to high-density lipoprotein (HDL) has been found to contain the amino acid products of these genes. On the other hand, *PON2*, which was found in a range of tissues, had the highest lactonase activity [192].

*B. cereus* produces AHL-lactonase enzymes, which regulate the virulence factors that the opportunistic *P. aeruginosa* produces, including pyocyanin and biofilm formation [193].

*A. acidoterrestris* thermoacidophilic bacteria generates QQ lactonase, which has a wide substrate display and a high hydrolyzing efficiency for both short- and long-chain AHLs [194]. Furthermore, lactonase isolated from *Muricauda olearia* inhibits the virulence factors in a range of pathogenic bacteria by degrading both long- and short-chain AHL [195].

*P. aeruginosa* poses a health risk to the general public by forming biofilms in potable water systems. Liu et al. examined the QQ activity of lactonase analog AiiADH82, which was isolated from the marine bacterium *B. velezensis*, against *P. aeruginosa* which was isolated from the water pipeline [196]. The findings showed that *P. aeruginosa*’s early proliferation, biofilm formation, and production of virulent factors were significantly inhibited by AiiADH82 [196].

Several bacterial spp. were revealed to have reduced virulence factors and efficient suppression of biofilm formation by an AHL-lactonase, which is encoded by aiiA of *Bacillus* spp. and belongs to the Metallo-β-lactamase superfamily [197,198].

#### 7.9.2. Acylase Enzyme

AHL molecules serve as the only source of energy and nitrogen for the acylase enzyme, so it breaks down the amide bond between the fatty acid side chain and lactone ring (Figure 3) [199]. The first microbe known to manufacture the acylase enzyme is *Variovorax paradoxus*. The acylase enzyme was also generated by the kidney in porcine subjects, *Pseudomonas* strain PAI-A, *P. aeruginosa* PAO1, and *Rhalstonia* strain XJ12B [200,201,202]. Since acylases, like *Pseudomonas*’ acylase PvdQ, can recognize acyl chains, they have greater substrate selectivity than lactonases [203].

#### 7.9.3. Oxidoreductase Enzyme

Unlike acylase and lactonase enzymes, which break down the acyl chains of AHLs, oxidoreductase enzymes reduce or oxidize them (Figure 3). This prevents AHL receptor binding and further QS-regulated gene expression [204].

### 7.10. Active Uptake of AI Signaling Molecules by Beneficial Bacteria

Other bacteria, like those in the Enterobacteriaceae family, can sequester and interfere with cell communication as competitors. *S. meliloti*, *S. typhimurium*, commensal *E. coli* K12, *B. anthracis*, and *E. coli* O157 are some of these bacteria [205]. Commensal bacteria remove AI-2 from the environment, so other members will no longer be able to use AI-2 signals to manipulate their behavior. It was reported that the bioluminescence caused by QS signals was reduced by 18% when *E. coli* was co-cultured with *V. harveyi* and inhibited by 90% when it was co-cultured with a mutant strain of *E. coli* that contains a constitutively inhibited LsrK [205].

## 8. Applications on QSIs in Combating Bacterial Biofilm Formation

A biofilm is a network made up of thousands of bacteria that serve as a defense against antibiotics, harsh environments, and the human immune system. Bacteria can develop as free-floating planktonic organisms or sessile, as in biofilms, which are structured from colonies enclosed in an extracellular polymeric matrix (EPS). The EPS, which is mostly made up of carbohydrates, lipids, proteins, and nucleic acids, makes up around two-thirds of the volume of the biofilm and provides the bacteria with a 3D protective framework. Incorporated bacteria inside the matrix can collaborate, exchange information, and transfer resistance genes. Lower oxygen and nutrient levels in the deeper matrix can create latent persisting cells that promote antibacterial tolerance and resistance. Treatment for MDR biofilm infections is particularly difficult since the physical barrier that biofilms create must be broken down to combat the infection [28,206,207,208,209,210].

Using QSIs in combating and destroying biofilm infection is a significant approach, and several modern studies demonstrated their promising effect. For example, in a mouse model of pulmonary infections, the acylase PvdQ is a QS-inhibiting agent with great therapeutic efficacy that can hydrolyze AHL signaling molecules irreversibly [211].

Furthermore, infections brought on by biofilm formation on medical devices continue to be a serious clinical issue because biofilms are one of the key virulence factors of pathogenic bacteria [212]. Thus, the formation of biofilm can be inhibited using a poly(ethylene glycol)-based multifunctional coating that permits the covalent incorporation of the synthetic QS inhibitor, 5-methylene-1-(prop-2-enoyl)-4-(2-fluorophenyl)-dihydropyrrol-2-one, on its surface [212]. This offers a practical means of avoiding infections linked to devices. In addition, QS-inhibiting drugs can be used as antibiotic accelerators to treat infections caused by bacteria. It was discovered that two cinnamic acid derivatives, 4-dimethylaminocinnamic acid and 4-methoxycinnamic acid, which function as AHL inhibitors, not only significantly inhibit the formation of biofilms but also increase their susceptibility to tobramycin [213].

In *A. baumannii*, biofilm production is dependent on the stimulation of a LuxI/LuxR-type QS system that includes AbaI synthase, AbaR receptor, and numerous AHLs [214]. Mayer et al. [215] observed that the mutation of the AHL synthase AbaI alters the surface-associated motility and persistent biofilm formation in *A. baumannii*. AHL analogs, anoR antagonists (like virstain), AbaR antagonists (like streptomycin), and antagonists for the di-guanylate cyclase enzyme—which produces cyclic di-GMP—were also shown to impede QS, which in turn prevented *A. baumannii* and *A. noscomialis* from forming biofilms [216,217,218,219]. Allam et al. [220] isolated siphonocholin, marine steroid, from *Siphonochalina siphonella* and discovered that this isolate possesses QSI activity and suppresses EPS synthesis, swarming motility, and biofilm formation in *A. baumannii*, MRSA, and *P. aeruginosa*. Lin et al. [221] evaluated the QS inhibiting activity of glabridin, a major bioactive component present in licorice flavonoids, against multi-drug resistant *A. baumannii*. They discovered that glabridin is a strongly anti-virulent agent that can inhibit biofilm development through the downregulation of QS-related genes, *abaI* and *abaR*, in *A. baumannii*. Chow et al. [222] developed a genetically engineered QQ lactonase and found that this enzyme can destroy the biofilm structure of *A. baumannii* via biomass reduction.

Several studies reported the effectiveness of QSIs in eradicating *P. aeruginosa* biofilm [84]. Various natural [223,224,225,226,227,228,229,230] and synthetic [140,231,232,233,234,235,236,237] QSIs were reported to possess the ability to destroy biofilm structure and help in eradicating resistant pathogens. Soukarieh et al. [238] conducted a novel approach by loading QSIs on the polymer to enhance their penetration and sustain their action. The authors discovered that a polymer–QSI conjugate may successfully penetrate biofilm layers and release the QSI. Additionally, when this conjugate was combined with ciprofloxacin, it increased the biofilm antibacterial activity of this antibiotic in comparison to free QSI and ciprofloxacin under the same circumstances. Al-Saafin et al. [239] conducted a novel approach using probiotic (*Lactobacillus plantarum*), prebiotic (Fructooligosaccharides) or a combination of them in reducing and inhibiting virulence and biofilm formation on *P. aeruginosa*. The authors discovered that *L. plantarum* especially can also affect bacterial metabolomics profiles. Sharma et al. [240] found that the treatment of *P. aeruginosa* biofilm with DNase I was highly effective in disrupting biofilm formation and destroying preformed biofilm.

Liu et al. [241] demonstrated that tea polyphenols possess anti-QS activity against *K. pneumoniae* and produce significant results in vivo and in vitro. An in vitro investigation revealed that tea polyphenols had anti-QS action against *C. violaceum.* Tea polyphenols at sub-MICs prevented motility and decreased the synthesis of protease and EPS, in addition to inhibiting biofilm formation. Further, an in vivo investigation found that tea polyphenols enhanced *Caenorhabditis elegans* survival versus *K. pneumonia* infection.

Meredith et al. [242] found that a synthetic QSI, N-phenyl-4-(3-phenylthioureido) benzenesulfonamide, allosterically alters the AI-3 receptor, inhibiting pathogenicity activation and reducing biofilm development in *E. Coli*. Then, Vinothkannan et al. [243] found that fructose furoic acid ester isolated from *Melia dubia* plant possesses QSI activity against *E. Coli* biofilm. They discovered that fructose furoic acid ester competes with the SdiA native ligand C8HSL to downregulate its target-specific expression and behavior.

Sully et al. [156] discovered a small molecule inhibitor, savirin (*S. aureus* virulence inhibitor), that blocked agr-mediated quorum sensing in *S. aureus*. According to their findings, savirin targets AgrA to disrupt agr operon-mediated QS. Rajendran et al. [244] demonstrated that naturally occurring flavonoids extracted from *Scutellaria oblonga* plant can reduce biofilm matrices versus *S. aureus*, *B. subtilis*, and *E. coli* by 73.5, 88.9 and 75.5%, respectively.

Rukayadi et al. extracted xanthorrhizol from the rhizome of *Curcuma xanthorrhiza* Roxb and demonstrated that xanthorrhizol can significantly reduce *S. mutans* biofilm by 76% after 1 h of treatment.

These are some of the few studies that have been reported in [245] to use QSIs in the management of bacterial biofilm formation.

## 9. Polyphenols as QSIs

Natural and dietary components with QSI properties have been the subject of recent research [146]. Many phenol units combine to form the large family of naturally occurring compounds known as polyphenols, which are mostly found in plants. Their antimicrobial and antifouling properties were reported [246,247,248,249]. The initial proof that polyphenolic compounds may disrupt bacterial QS was demonstrated by a study that used *P. putida* and *E. coli* as AHL biosensors to test the QS inhibitory activity of different polyphenolic compounds with gallic acid moiety [248].

Subsequent research focused on screening various polyphenols according to their QS inhibitory activity because they are easily isolated and abundant in natural resources [249,250]. For example, methyl gallate was employed as an inhibitor to combat *C. violaceum*, which can cause fatal sepsis and cause liver and lung problems. This bacterium is widely used to investigate the inhibition of acyl-homoserine lactone-dependent QS by various compounds because it produces violacein pigment in response to QS-regulated gene expression [251]. Green tea polyphenols were also discovered to exhibit QS inhibitory action against *P. aeruginosa* both in vivo and in vitro [250].

*Rosa rugosa* contains around 87% polyphenols, which make up the majority of the plant’s extracts. When *Rosa rugosa* was evaluated for QSI activity using *C. violaceum* 026, it considerably lowered the quantity of violacein generated without inhibiting the growth of the bacteria. In addition, phenols in *rosa rugosa* tea significantly inhibited the swarming motility and biofilm formation of *P. aeruginosa* PAO1 and *E. coli* K-12 [252].

## 10. Novel Approaches Using QSIs to Combat Resistant Bacteria

### 10.1. QSIs-NPs

Recent developments in nanobiotechnology allowed for the development of novel QSI products and formulations with improved therapeutic potential, tailored delivery, and reduced toxicity. Numerous beneficial properties of nanomaterials include their large surface area, small molecular size, biocompatibility, and generally controlled toxicity [253]. Due to their high reactivity, nanomaterials have been applied in a wide range of biological applications. Numerous applications of nanotechnology in medicine have given rise to a new field called “nanomedicine”. Research is moving now towards alternatives as a result of the growing problems with antibiotic resistance [116].

**Figure 4:**
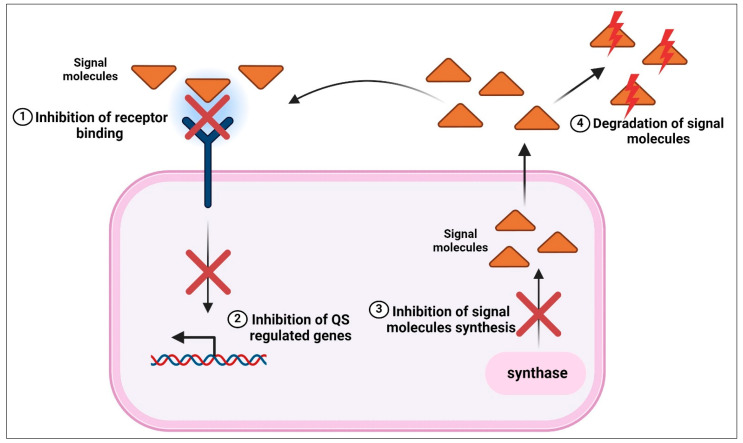
Schematic illustration of the possible ways by which NPs could obstruct the QS process. Created with BioRender.

Metal NPs have great potential as strong antibacterial agents. When they get inside bacterial cells, they may damage the bacterial cell membrane [254,255,256,257]. Recent in vivo and in vitro studies revealed that metal NPs can inhibit the QS system. The role of NPs as QSIs or as carriers of QQ materials has been documented in certain reports, despite a lack of literature on the subject. Signaling molecules can be inhibited from degrading, synthesizing, or binding to their receptors, which can all cause QS inhibition [116]. How NPs can interfere with QS systems is explained in a simplified diagram (Figure 4).

Numerous studies have employed silver NPs (AgNPs) as antimicrobial agents and reported their antibiofilm activity and broad-spectrum activity against a variety of bacteria, including *S. aureus*, *P. aeruginosa*, and *E. coli* [258]. AgNPs may prevent the production of AIs by preventing LasI/Rhl I synthase, which subsequently prevents *P. aeruginosa*’s QS [172]. According to published research, AgNPs are effective anti-QS agents that can prevent *C. violaceium* from forming biofilms and producing violacein [259].

Yttrium oxide (Y_2_O_3_) NPs are another type of nanomaterial that is utilized in biomedicine. Due to the physiochemical characteristics of Y_2_O_3_NPs, there is increasing interest in the use of these materials in biomedical applications [260]. Husain et al. created spherically shaped monodispersed core and core/shell nanospheres (NSs) of Y_2_O_3_ and evaluated its capacity to inhibit AHL in *C. violaceum* CVO26 and *P. aeruginosa* PAO1 [253]. The results showed core and core/shell NSs inhibit the QS-controlled virulent factors in *P. aeruginosa* such as the formation of exopolysaccharides, pyocyanin, elastase, protease, and swarming motility.

Moreover, Ravindran et al. synthesized AgNPs from the root extract of *Vetiveria zizanioides*. The authors found that the synthesized AgNPs downregulate the QS-regulated genes in *S. marcescens* and reduce the production of biofilm, lipase, protease, and exopolysaccharide as well as other QS-dependent virulence factors, without affecting bacterial growth [261]. Other NP forms from microbial sources, such as AuNPs, TiO_2_, SiO_2_, and ZnO, can effectively block the QS cascade and inhibit the development of the biofilm [262,263,264].

Research also showed that *Proteus* sp. QS is inhibited by the acyl-homoserine lactone lactonase protein linked to Au NP [265]. With the assistance of N-acyl homoserine lactonase, which was present on the NPs’ surface, these NPs were also capable of breaking down N-hexanoyl-L-homoserine lactone. The enzyme also aids in the breakdown of the acyl-homoserine lactone moiety and alters the signaling molecule’s conformation to prevent binding with the LuxR transcriptional regulator, thus inhibiting QS [266,267]. Additionally, they could stop EPS synthesis and metabolic processes, which prevented the bacterial cells from forming biofilms and altered their hydrophobicity [263]. The stabilization of NPs was achieved by the Au NP generated from the mycelium of *Laccaria fraternal*, which in turn plays a significant role in the reduction in pyocyanin production from *P. aeruginosa* [263].

AgCl-TiO2 NPs were shown to be an efficient anti-QS agent against *C. violaceum* in experimental observations [268]. Studies revealed that β-cyclodextrin-coated NPs aid in the suppression of *V. fischeri*’s AHL-dependent QS, the results of the study demonstrated that the presence of β-cyclodextrin linked to Si-NP aids in the removal of AHL molecules from the surrounding environment and decreases bioluminescence. Furthermore, it was found that these NPs could downregulate the *LuxA* and *LuxR* genes [269].

Numerous studies that have been conducted have shown how effective ZnO NPs are as an anti-QS agent. In a *P. aeruginosa* strain isolated from cystic fibrosis, biofilm formation was significantly influenced by the inhibition of the QS mechanism [270]. These nanoparticles can suppress the expression of QS genes in Gram-negative bacterial cells [271]. ZnO NPs inhibited QS in *P. aeruginosa* by downregulating *LasR*, *LasI*, *Rhl I*, and *Rhl R*, according to research findings [272]. ZnO NPs have been demonstrated in another study to be effective against the QS PQS and Las system as well as to be able to decrease the swimming and swarming motility of *P. aeruginosa* [273]

Interestingly, a combination of NPs and anti-bacterial agents was reported to possess a synergistic effect against the QS system. For example, QS-regulated bacterial virulence can be inhibited by chitosan NPs loaded with kaempferol, a naturally occurring flavonoid found in many plants [274]. In this investigation, the biosensor strain of *C. violaceum* CV026 was used to assess the inhibitory ability of the chitosan-loaded NPs using the violacein pigment-producing assay. The authors concluded that kaempferol can inhibit QS when loaded on chitosan with a concentration exceeding 78%.

### 10.2. Combination of QSIs with Traditional Antibiotics through Linkers

**Figure 5:**
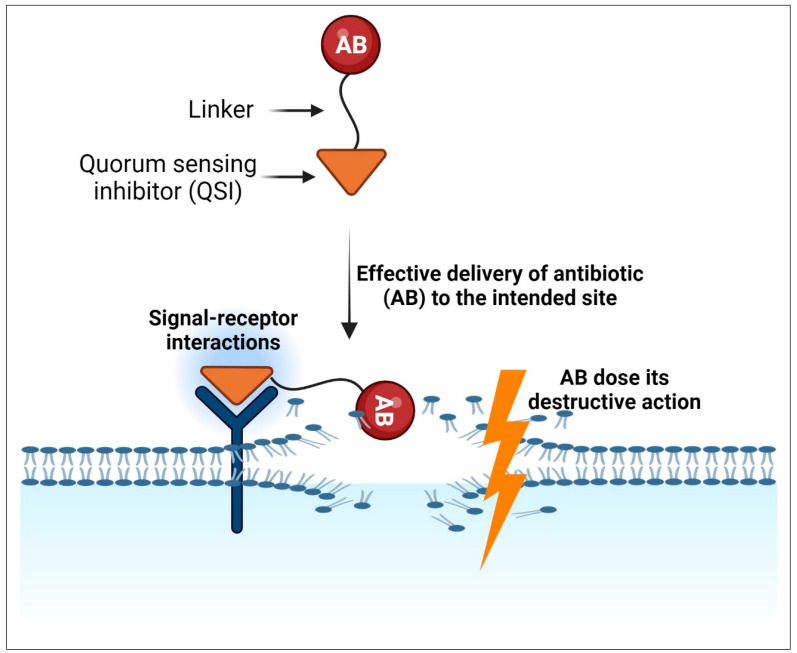
Diagram demonstrating the QSI-guided antibiotic delivery. Modified from [277]. Created with BioRender.

Even though QSIs possess a therapeutic promise, they do not have an impact on bacterial cell viability [84,187,275]. So, the combination of QSIs with traditional antibiotics is an effective strategy to achieve clinically successful therapy (Figure 5). One effective tactic is to employ the QSIs as a guiding or delivery vehicle, to which any antibiotic may be linked using the appropriate linkers to ensure effective delivery to the intended site. This formulation can recognize the target and accumulate on the cell membrane via signal-receptor interactions. The combination will offer several advantages, including decreased virulence due to QS regulation, increased antibacterial activity, selective bacterial death, less off-target toxicity, and killing bacteria with a lower antibiotic dosage [276].

Numerous linkers have been used, such as cleavable hydrazone, disulfide, or peptide linkers, as well as non-cleavable thioether or maleimidocaproyl linkers [278]. Non-cleavable linkers can decrease medication release and effectiveness, although they offer higher stability and tolerability. It has been demonstrated that cleavable linkers composed of disulfide or hydrazone bonds are susceptible to reductive or oxidative conditions, respectively, and that these conditions may occur outside of cells. Thus, several linker-targeted enzymes are found around the site of infection to preferentially break the linkers. On the other hand, peptides are excellent enzyme-cleavable linkers since many hydrolytic enzymes can identify their sequences [279,280].

In this case, the QSI will prevent bacterial communication and concurrently direct the antibiotic toward the targeted bacteria. This prevents bacteria from developing virulent factors, while the antibiotic kills the bacteria at the site of infection.

Brackman et al. [281] reported that a combined therapy improved the survival rates for *Galleria mellonella* larvae and *C. elegans* infected with a *P. aeruginosa* and *B. cepacia* complex. The authors discovered that the bacterial burden in the lungs of BALB/c mice infected with *B. cenocepacia* was decreased by combining tobramycin with cinnamaldehyde when compared to using antibiotics alone. Furthermore, Yu et al. [282] demonstrated that the combination of sulfonamide antibiotics, Ag antimicrobial agents such as AgNPs, and Ag nitrate with QSIs potentiates the anti-microbial activity against *B. subtilis*. Singh et al. [283] demonstrated that the dual bioresponsive ciprofloxacin and QSI, 3-amino-7-chloro-2-nonylquinazolin-4(3H)-one, combined with alginate NPs, effectively destroy mature *P. aeruginosa* biofilm in vitro and ex vivo. The authors created a bioresponsive polymer formulation using specially altered alginate nanoparticles to deliver ciprofloxacin in conjunction with the tested QSI. They also engineered alginate NPs and incorporated a pH-sensitive linker between the polysaccharide backbone and QSI molecules. By this way, QSI linker cleaved on low PH biofilm regions, enabling the concomitant release of ciprofloxacin and QSI. With this dual-action bio-responsive ciprofloxacin and QSI release, NPs efficiently eradicated *P. aeruginosa* infection in an ex vivo skin infection model.

### 10.3. Repurposing of Previously Known Drugs as QSIs

Drug repurposing (DR) is also referred to as drug re-tasking, recycling, reprofiling, rescue, redirection, and medical switching. This process involves identifying new pharmacological indications in already marketed or FDA-approved pharmaceuticals [284]. In the means of combating antibiotic resistance, DR means redirection and switching of already existing non-antibiotic drugs to act against resistant pathogens like antibiotic drugs. This scenario can bridge the gap in discovering novel antibiotic candidates [285,286,287].

Amazingly, Abbas et al. [288] demonstrated that metformin, an anti-diabetic drug, can act as a QSI against *P. aeruginosa*. The authors found that metformin greatly reduced violacein pigment production, blocked pyocyanin, hemolysin, protease, and elastase, and markedly decreased biofilm formation and the motility of *P. aeruginosa* via binding to *LasR* and *RhlR* receptors. Then Hegazy et al. [289] found that metformin had considerable anti-QS activity in vitro but did not protect mice against *P. aeruginosa*. Conversely, sitagliptin, another anti-diabetic drug, decreased the virulence of *P. aeruginosa* in vitro and protected mice against this pathogen. Most recently, Khayat et al. [290] discovered that both metformin and vildagliptin significantly downregulate the expression of the *P. aeruginosa* genes that code for QS and a combination of these two anti-diabetic drugs can significantly decrease the development of biofilms, bacterial motility, and the generation of virulent extracellular enzymes and pyocyanin pigment. Additionally, it significantly increases *P. aeruginosa*’s vulnerability to oxidative stress. In another study by Khayat et al. [291], 10 gliptins were evaluated for their QSI activity in vivo and in vitro. They demonstrated that all tested gliptins, especially sitagliptin, possess anti-biofilm activity. Additionally, they found that sitagliptin effectively protects mice from *S. aureus* and *P. aeruginosa* pathogenesis. Moreover, they reported that QS-encoding genes in *S. aureus* and *P. aeruginosa* were downregulated by sitagliptin.

Ho Sui et al. [292] discovered that raloxifene, which was originally indicated for breast cancer, bonds to PhzB2 in *P. aeruginosa*, inhibiting the formation of blue pigment pyocyanin, a key factor in infection. Imperi et al. [293] showed that niclosamide, an old anthelmintic drug, significantly reduced the generation of AHL molecules and thus QS response in *P. aeruginosa*. The authors concluded that niclosamide possesses a strong anti-virulence activity in vitro and protects against *P. aeruginosa* pathogenicity in vivo.

Singh et al. [294] found that albendazole has significant potential as a QSI. It inhibits QS by binding with hydrophobic amino acid residues in the hydrophobic pocket of LasR and CviR receptors in *P. aeruginosa* and *C. violaceum*, respectively. So, it can inhibit biofilm formation in *P. aeruginosa* and violaceum production in *C. violaceum*.

## 11. Clinical Trials

As stated above, several studies conducted on a lab scale have demonstrated the promising effect of QSIs against several bacterial pathogens. There are few clinical trials that evaluate their effectiveness in combating bacterial virulence.

In a clinical trial by Zhu et al. [295], the safety of furanone or fimbrolides was evaluated in human volunteers’ and guinea pigs’ contact lenses against *P. aeruginosa*, *S. aureus*, *S. marcescens*, and *Acanthamoeba* spp. The efficacy of contact lenses coated with fimbrolides against bacterial adhesion was established throughout a trial period spanning 30 days for animals and 24 h for humans. Bacterial adhesion of all tested strains was shown to be reduced by 67–92%, which supports their usage as antipathogens. Additionally, the authors reported that the ocular response was not affected by the usage of coated lenses during the trial period, which demonstrates their safety.

Another clinical trial by Smyth et al. [296] examined the effect of garlic formulation as QSI in children and adults with cystic fibrosis and persistent infection with *P. aeruginosa*. The garlic formulation failed to produce substantial enhancement in clinical parameters, and there was no decrease in the amounts of QS molecules neither in plasma nor sputum samples. Furthermore, some patients experienced abnormal liver function, and some had mild side effects; overall, the study was revolutionary and encouraged more studies.

In a randomized placebo–control trial by van Delden et al. [297], the effect of azithromycin, a macrolide antibiotic with QSI activity, was evaluated for ventilator-associated pneumonia (VAP) in intubated patients colonized with *P. aeruginosa*. The author found that azithromycin dramatically reduced VAP in individuals with a high risk of rhamnolipid-dependent VAP, indicating that virulence suppression is a viable anti-microbial approach. Fong et al. [298] discovered that itaconimides possess QSI activity by suppressing *Las*, *Rhl*, and PQS QS systems of *P. aeruginosa*. The authors reported that itaconimides effectively eliminate virulent activities. Additionally, they observed a synergetic activity of itaconimides when combined with tobramycin.

## 12. Conclusions

Research on the QS system has shown that bacteria have developed a variety of intra- and interspecies communication mechanisms. It is crucial to develop new strategies to combat MDR bacteria. In recent decades, numerous biomedical studies have focused on QS-mediated infectious diseases and the potential use of QSIs to combat the growing problem of antibiotic resistance. In adverse conditions, biofilms can help bacteria survive, because biofilms’ extracellular matrix prevents conventional antibiotics and bactericides from penetrating, and bacteria are less sensitive to them. Since the QS system controls how a biofilm forms, using QSI agents is a potentially effective way to manage the formation of biofilms. Further investigations are necessary to understand how QS signaling molecules regulate antimicrobial resistance in clinical isolates. To combat microbial resistance, new QQ molecules will be introduced as a result of innovation and advancement in research on QQ molecules and their effects on regulatory systems. Over the next several decades, there will likely be a significant increase in the discovery of QQ molecules. These molecules may be employed in taking the place of bacteriostatic and bactericidal antibiotics in vivo.
